# Trends in presentation, management and survival of patients with *de novo* metastatic breast cancer in a Southeast Asian setting

**DOI:** 10.1038/srep16252

**Published:** 2015-11-05

**Authors:** Nirmala Bhoo-Pathy, Helena Marieke Verkooijen, Ern-Yu Tan, Hui Miao, Nur Aishah Mohd Taib, Judith S. Brand, Rebecca A. Dent, Mee-Hoong See, ShriDevi Subramaniam, Patrick Chan, Soo-Chin Lee, Mikael Hartman, Cheng-Har Yip

**Affiliations:** 1 Julius Centre University of Malaya, Centre for Evidence Based Medicine and Clinical Epidemiology, Department of Social and Preventive Medicine, Faculty of Medicine, University of Malaya, 50603 Lembah Pantai, Kuala Lumpur, Malaysia; 2 Julius Center for Health Sciences and Primary Care, University Medical Center, PO Box 85500, 3508 GA Utrecht, The Netherlands; 3 Saw Swee Hock School of Public Health, National University of Singapore, MD3, 16 Medical Drive, Singapore 117597; 4 Department of General Surgery, Tan Tock Seng Hospital, 11 Jalan Tan Tock Seng, Singapore 308433, Singapore; 5 Department of Surgery, Faculty of Medicine, University of Malaya, 50603 Lembah Pantai, Kuala Lumpur, Malaysia; 6 Institution of Medical Epidemiology and Biostatistics, Karolinska Instituet, Nobels Väg 12A, 171 77 Stockholm, Sweden; 7 National Cancer Centre Singapore, 11 Hospital Dr, Singapore 169610; 8 Duke-NUS Graduate Medical School Singapore, 8 College Road, Singapore 169857; 9 National Clinical Research Centre, Level 3, Dermatology Block, Kuala Lumpur Hospital, 50586 Jalan Pahang, Kuala Lumpur, Malaysia; 10 Department of Hematology Oncology, National University Cancer Institute, National University Health System, Singapore 119228.

## Abstract

Up to 25% of breast cancer patients in Asia present with *de novo* metastatic disease. We examined the survival trends of Asian patients with metastatic breast cancer over fifteen years. The impact of changes in patient’s demography, tumor characteristics, tumor burden, and treatment on survival trend were examined. Patients with *de novo* metastatic breast cancer from three hospitals in Malaysia and Singapore (N = 856) were grouped by year of diagnosis: 1996–2000, 2001–2005 and 2006–2010. Step-wise multivariable Poisson regression was used to estimate the contribution of above-mentioned factors on the survival trend. Proportions of patients presenting with metastatic breast cancer were 10% in 1996–2000, 7% in 2001–2005, and 9% in 2006–2010. Patients in 2006–2010 were significantly older, appeared to have higher disease burden, and received more chemotherapy, endocrine therapy, and surgery of primary tumor. The three-year relative survival in the above periods were 20·6% (95% CI: 13·9%–28·2%), 28·8% (95% CI: 23·4%–34·2%), and 33·6% (95% CI: 28·8%–38·5%), respectively. Adjustment for treatment considerably attenuated the relative excess risk of mortality in recent years, compared to other factors. Substantial improvements in survival were observed in patients with *de novo* metastatic breast cancer in this study.

Metastatic breast cancer is an incurable disease where treatment strategies aim to achieve disease control, improve quality of life, and attain clinically meaningful prolongation of survival^1^. A number of studies conducted in affluent Western settings have shown improvement in survival of patients with *de novo* metastatic breast cancer^2^,^3^,^4^,^5^.

Approximately 10 to 25% of breast cancer patients in Asian countries present with *de novo* metastatic disease, compared to only 3 to 5% in Europe and United States^6^,^7^,^8^,^9^. Besides a high burden of patients presenting with distant metastases at initial diagnosis in Asian settings, the profile of disease in these patients is more severe whereby higher proportions of locally advanced tumors are seen and distant metastases are mostly detected due to symptoms and are more likely to involve multiple metastatic sites^10^.

While it is expected that improving awareness of breast cancer in the population, and development of health-care systems in Asia might influence earlier detection of breast cancer^11^,^12^, it is unknown whether the situation in *de novo* metastatic settings have changed at all in Asia.

We examined the patterns in disease presentation, management, and survival of women presenting with *de novo* metastatic breast cancer over a 15-year period in a multi-ethnic Asian setting. Respective impact of changes in demography, tumor burden, tumor characteristics, and treatment on the survival trend were assessed.

## Methods

### Ethics approval

This study obtained ethics approval from the University Malaya Medical Centre’s Ethics Committee and the National Healthcare Group (NHG) Domain Specific Review Board (DSRB). The study methods were carried out in accordance with approved guidelines. As the study relies on non-identifiable registry-based data, the need to obtain informed consent was waived.

### Study population and setting

This study includes 856 women who were diagnosed with *de novo* metastatic breast cancer between 1996 and 2010, in University Malaya Medical Centre (UMMC; 445 patients), Malaysia, National University Hospital (NUH; 185 patients), Singapore, or Tan Tock Seng Hospital (TTSH; 226 patients), Singapore. All three hospitals are tertiary referral centres, with prospective breast cancer registries since 1995 in UMMC and NUH, and 2001 in TTSH^8^,^13^.

Staging of primary breast cancer consisted primarily of physical examination, chest x-ray, and liver ultrasound. In all centers, targeted metastatic work-up by means of computed tomography scan of thorax, abdomen +/− pelvis and bone scans were performed in symptomatic patients, and women with high nodal burden and large tumors. Routine biopsy of metastasis was not performed in any of the centers.

Patients presenting with only ipsilateral supraclavicular lymph node involvement were excluded from the current study.

### Study variables

Study variables encompass age at diagnosis, ethnicity, and primary tumor characteristics; tumor size at presentation, histology, estrogen receptor (ER) status. Tumor grade was missing in more than 50% of patients. Since testing for human epidermal growth factor receptor 2 (HER2) status was only routinely done after 2005 in all centers, this variable was not included in analysis. Information on distant metastases included number of metastatic sites, and site of metastases. Patients having liver, and/or brain, and/or lung involvement were classified as having visceral metastases, whereas those with bone, and/or, skin, and/or lymph nodes (other than axillary) metastases but without visceral involvement were classified as not having visceral metastases.

Variables on treatment comprise chemotherapy, endocrine treatment, surgery of primary tumor, surgical margins, and radiotherapy of breast, chest wall or other metastatic sites. Information on exact chemotherapy regimen or targeted treatment was only sparsely available and not included in this study.

### Follow-up and outcome assessment

Data on all-cause mortality was updated through direct linkage with the respective National Registration Departments of Malaysia, and Singapore, which hold the birth and death records of all nationals and cover the entire populations, as it is mandatory to register births and deaths in both countries. Follow-up was calculated from the date of diagnosis of *de novo* metastatic breast cancer, to the date of death or censored at end of follow-up (February 2012 in UMMC, July 2012 in NUH patients, and October 2012 in TTSH).

### Statistical Analysis

Patients were assigned to three equal time groups of five-year intervals based on their year of diagnosis, i.e. 1996–2000, 2001–2005, as well as 2006–2010. The proportion of patients with *de novo* metastatic breast cancer from the overall breast cancer patient population was determined according to the above periods. Within patients with *de novo* stage IV breast cancer, demographic characteristics, clinical presentation, tumor profile and treatment patterns were compared across the periods using Chi square test for categorical variables, and Kruskal Wallis test for continuous variables.

As cause of death was largely not available, and it had been previously shown that overall survival differs significantly from breast cancer-specific survival even in stage IV breast cancer patients^2^, relative survival rates (RSR) were estimated^14^. Relative survival is the ratio of all-cause survival observed in patients with metastatic breast cancer to the survival that would have been expected had they been subjected only to the mortality rates of the general population. It can be interpreted as net survival attributable to (metastatic breast) cancer. Expected survival was derived from life tables that contained the probabilities of death for the general population in Malaysia and Singapore, by age, gender, country, and single calendar year between 1996 and 2012.

To adjust survival rates for differences in center of treatment, follow-up time, patients’ age, ethnicity, tumor T stage at diagnosis, ER status, number of organs with metastatic involvement, presence of visceral metastasis, surgery, surgical margins, radiotherapy, chemotherapy, and endocrine therapy between the three periods, relative excess risks (RER) was modelled using multivariable Poisson regression^15^. The advantage of using RER is that it takes into account the background risk of death in the general population^15^, therefore adjusting for any systematic differences in the mortality rates between Malaysia and Singapore. In this study, it maybe interpreted as the relative risk of mortality from metastatic breast cancer in a given period compared to the reference period (1996–2000).

The Poisson model was adjusted in a stepwise approach for demography, disease characteristics, and treatment, to gauge the individual impact of these factors towards the observed changes in survival throughout the three time periods.

Missing values namely primary tumor size (29%), estrogen receptor status (16%), number of organs with distant involvement (9%), visceral involvement (9%), surgical margin status (20%), chemotherapy status (9%), radiotherapy status (24%), and endocrine therapy status (17%) were imputed by means of multiple imputation^16^ using the ICE package in STATA^17^. All variables of the multivariable adjusted model were included in the imputation model and 10 imputation sets were created.

Data was analyzed using STATA version 12.0 (Stata Corp., College Station, TX, USA).

## Results

Proportion of patients presenting with *de novo* metastatic breast cancer from the overall breast cancer cases remained stable over the three time periods (10% in period 1, 7% in period 2, and 9% in period 3). Patients presented at a median of 53 years and comprise Chinese (60%), Malays (27%), Indians (11%) and other races (3%). Approximately 70% of patients presented with tumor sizes of 5cm or more, while 50% of patients had metastasis involving more than one organ site. Visceral metastases were found in a majority of patients (87%). Eighty-two patients (~10%) did not receive any form of treatment. Median survival was 18 months (95% CI: 16–20 months).

Compared to patients diagnosed in period 1, those diagnosed in period 3 were older and less often of Malay descent (Table 1). There was no significant change in proportion of patients presenting with primary tumors measuring more than 5cm, in recent periods (Table 1). Nevertheless, it was found that in women aged <= 50 years, proportion of tumors measuring less than 5cm increased from 12% to 24% between period 1 and period 3 (p = 0.048), whereas in the older women this proportion only increased from 30% to 34% (p = 0.730). Patients who were recently diagnosed were more likely to have multiple organ sites involvement and visceral metastases compared to women diagnosed in earlier periods.

Approximately 90% of tumors were ductal carcinomas in all three periods. Estrogen receptor was expressed in 62% of patients in period 2, and 59% of patients in period 3, compared to only 53% in period 1 (53%); *p* = 0.469.

In the first period, only about 55% of patients received chemotherapy, whereas 45% of patients with hormone receptor positive tumors received endocrine therapy (Table 2). The proportions of patients receiving systemic therapy increased over time, albeit not statistically significant for endocrine therapy. The chemotherapy administration rates for instance increased by close to 10% (to 64%), whereas endocrine therapy administration increased by 15 per cent (to 60%), between period 1 and period 3. Patients diagnosed in recent years were also significantly more likely to undergo surgery of the primary tumour, and attain free surgical margins.

**Figure 1:**
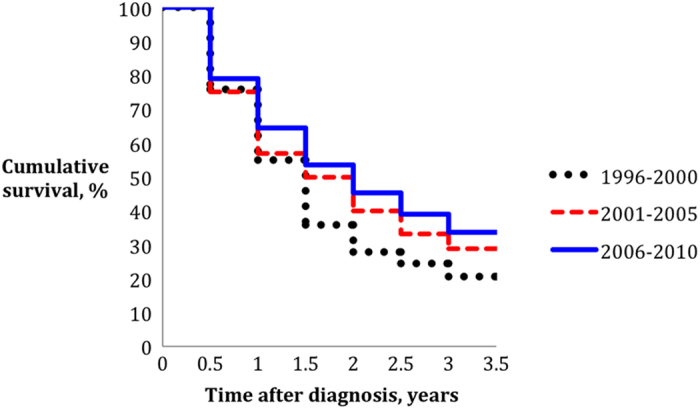
Cumulative relative survival by period of diagnosis in Asian patients with de novo metastatic breast cancer.

There were 665 deaths over 1644 person-years of follow-up. Median follow-up time was 1.39 years (25^th^ percentile: 0.58 years, 75^th^ percentile: 2.75 years). Eight patients were lost to follow-up. Median survival improved from 14 months (95% CI: 11–17 months) in period 1, to 18 months (95% CI: 13–22 months) in period 2, and 21 months (95% CI: 18–24 months) in period 3. The corresponding three-year relative survival rates in the above periods were 20.6% (95% CI: 13.9%–28.2%, median survival = 14 months), 28.8% (95% CI: 23.4%–34.2%, median survival = 18 months), and 33.6% (95% CI: 28.8%–38.5%, median survival = 21 months) (Fig. 1, Table 3). Patients in period 3 had a significantly higher survival than those in period 1; corresponding with a RER of 0.68 (95% CI: 0.54–0.86) (Table 4). To better understand the contribution of changes in patients’ demographic profile towards the survival gain, differences in age at diagnosis, and ethnic distribution, were first adjusted. This did not modify the RER for period 3 compared to period 1 (Table 4). Further adjustment for tumor characteristics (T stage, ER status), and distant disease burden (number of metastatic sites, and presence of visceral metastasis) only marginally changed the RER (from 0.68 [95% CI: 0.54–0.87] to 0.66 [95% CI: 0.52–0.84]). Adjustment for treatment brought about substantial attenuation in the RER for period 3. Initial adjustment for chemotherapy, and endocrine therapy, for instance attenuated the RER in period 3 to 0.71 (95% CI: 0.56–0.89), compared to period 1. Further adjustment for locoregional management; surgery of primary tumor, and surgical margins, resulted in a RER of 0.77 (95% CI: 0.61–0.98) for period 3 compared to period 1, whereas adjustment for radiotherapy (breast, chest wall, other metastatic sites) rendered the RER in period 3 statistically not significant; 0.79 (95% CI: 0.62–1.01).

## Discussion

In this study, we found that median survival of patients with *de novo* metastatic breast cancer rose from 14 months (95% CI: 11–17 months) to 21 months (95% CI: 18–24 months) over a span of fifteen years, which is largely attributed to improved treatment administration.

While a hospital-based study in France showed that the three-year survival rates of patients presenting with *de novo* stage IV breast cancer improved from 27% (median survival: 23 months) to 44% (median survival: 29 months) between 1987 and 2000^2^, a population-based study in the United States found that median survival in *de novo* metastatic breast cancer patients increased from 20 months to 25 months, between 1988 and 2003^3^. Our lower survival rates are most likely attributed to differences in disease spectrum^2^,^3^,^4^,^5^. In affluent Western settings, breast cancer patients are more likely to receive intensive work-up leading to detection of small and solitary metastatic lesions. In Asia, a substantial number of patients present with symptoms of metastatic disease, as well as higher proportions of locally advanced tumors, and multiple metastatic sites at initial diagnosis^10^,^18^. While access to modern cancer therapeutic agents may be responsible for the survival gain observed in previous studies, stage migration may also play a role; resulting from intensive screening to detect distant metastases, improved imaging facilities, or changes in diagnostic criteria. Only one small-scale study attempted to objectively examine impact of treatment on the trends of survival in patients with metastatic breast cancer. While the authors showed that receipt of aromatase inhibitors, and zoledronic acid were significant predictors of survival in patients with *de novo* metastatic disease, it was less clear whether these treatment contributed towards the improved survival trend^5^.

The increased availability of high quality imaging facilities in recent years may enable detection of cancer metastases before they become clinically evident, resulting in more patients who would have been previously classified as having non-metastatic breast cancer migrating to stage IV. This (Will Rogers) phenomenon^19^ may partly explain the (apparent) survival improvement of patients with *de novo* metastatic breast cancer. In the current study, the proportion of patients with *de novo* metastatic breast cancer out of the overall breast cancer patients was fairly stable over the three periods; 10%, 7%, and 9% respectively. It is hence unlikely that stage migration could completely explain the improved survival in our patients. However, it appeared that patients presenting with *de novo* metastatic breast cancer in recent periods were more likely to have higher number of involved metastatic sites, and visceral metastases. This actually reflects improvements in diagnostic facilities in the three centers, and perhaps higher tendency to screen for distant metastasis. However, adjustment for the above explained the survival gain observed in this study.

As patients with only ipsilateral supraclavicular lymph node metastases (i.e. stage IV breast cancer based on American Joint Committee on Cancer 5^th^ edition TNM system) were excluded, change in TNM stage classification is not a cause of concern in this study.

The changing age at presentation in this study reflects the demographic transition in our settings during the above periods^20^,^21^. It is conceivable that changes in ethnic distribution over time may be a reflection of differential metastatic work-up between different ethnic groups. We previously showed that Malay ethnicity is associated with poorer survival compared to other races, even after adjustment for late presentation, unfavorable tumor characteristics and sub-optimal treatment^22^. However, changes in demography had no impact on survival improvement of patients with *de novo* metastatic breast cancer in this study.

The proportion of women with tumors larger than 5 cm remained consistently close to seventy per cent throughout the two decades, particularly in women aged more than 50 years. This warrants intensification of early detection activities in these settings. Patients in recent times were also more likely to express ER positive tumors, which may be related to increasing age at diagnoses. Nevertheless, higher rates of ER positive tumors barely influenced the observed survival trends.

In this study, ten per cent of patients did not receive any form of treatment. In the first period, approximately fifty per cent of patients did not receive systemic therapy. This maybe a reflection of patient’s choice and not necessarily treatment policies, as it is not uncommon for Asian breast cancer patients to decline therapy or follow-up in order to seek alternative or traditional treatment^13^. While the systemic therapy administration rates in patients with *de novo* metastatic breast cancer in this study showed an absolute increase of up to 15% between period 1 and period 3, there remains room for improvement. For instance, a sizeable proportion of patients with hormone receptor positive tumors in period 3 were still not receiving endocrine therapy. The increase in rates of surgery of primary tumor in recent years may be influenced by findings of many observational studies which have shown that surgical removal of the primary tumor is associated with a survival benefit in patients with *de novo* metastatic breast cancer both in the Western, and Asian settings^10^,^23^. In this study, while it seems that adjustment for surgery of the primary tumor in the multivariable regression model brought about considerable attenuation in the relative excess risk of mortality for the most recent period, selection bias may pose a major challenge in estimating the ‘impact’ of surgical resection of primary tumor on survival of patients with *de novo* metastatic breast cancer. This is in view that patients who are generally fitter, with lower metastatic tumor load, better performance status^10^,^23^, and/or responding well to initial systemic therapy are more likely to have been selected to undergo surgery. Early results from two randomized controlled trials have in fact concluded that locoregional treatment do not confer any survival benefit in Asian patients with *de novo* metastatic breast cancer^24^,^25^.

Previous studies examining the survival trends in patients with *de novo* metastatic breast cancer were unable to disentangle the impact of various factors on the survival gain^2^,^3^,^4^,^5^. In these studies, the modest survival improvement in *de novo* metastatic settings were attributed to introduction of modern therapeutic agents including taxane-based chemotherapy, aromatase inhibitors, monoclonal antibodies, oral fluorouracil derivatives, and multiple novel agents targeting HER2 overexpressing tumors^2^,^3^,^4^,^5^. Our findings complements the findings of previous studies as it confirms that treatment indeed explains most of the survival gain in *de novo* metastatic settings. Interestingly, we have observed a higher margin of survival gain in the current study, which was largely attributed to the fact that more Asian patients with *de novo* metastatic breast cancer were receiving treatment in recent times.

Our finding is particularly pertinent not only in the Asian context but also in the light of global health. Cancer fatalism had been shown to be an important theme underlying non-participation in breast cancer screening, delayed presentation, and delayed treatment of breast cancer among the African Americans^26^, Latinas^27^, as well as Asians^28^,^29^. Non-acceptance and non-adherence of breast cancer treatment and follow-up measures among Asian women may also be attributed to lack of trust in the health system, and in one’s chances to survive^30^. The current study provides evidence for clinicians and patients alike, that although metastatic breast cancer is considered incurable, treatment had been largely responsible for improving survival in the setting of *de novo* disease, even in patients presenting at a more severe end of the disease spectrum as in Asia.

We do acknowledge that there are several limitations in this study. Firstly, we did not have any direct measures reflecting intensiveness of screening for metastatic lesions or improved imaging facilities. We had instead used reported tumor burden (number of involved metastatic sites, presence of visceral metastases) as proxy measurements. Detailed systemic treatment information was not available, which may have resulted in underestimation of the contribution of systemic therapy towards survival improvement. We also did not have data on administration of supportive/palliative care, as well as surgery of the metastatic sites^31^,^32^, which may affect prognosis of patients with *de novo* metastatic breast cancer. While we did not have data on tumor grade and HER2 status, which was only routinely assessed in period 3 in our centers (2006 and onwards), it is unlikely that tumor biology would have changed substantially over the three periods to have a meaningful impact on our study results.

Substantial improvements in survival were observed in patients with *de novo* metastatic breast cancer in this Asian setting over the last two decades. This survival gain was largely attributed to improvement in treatment administrations.

## Additional Information

**How to cite this article**: Bhoo-Pathy, N. *et al.* Trends in presentation, management and survival of patients with *de novo* metastatic breast cancer in a Southeast Asian setting. *Sci. Rep.* **5**, 16252; doi: 10.1038/srep16252 (2015).

## Figures and Tables

**Table 1:** Trends in Presentation of Asian Patients with *De Novo* Metastatic Breast Cancer.

Characteristics	Overall (856 patients) N, %	Period of diagnosis			P value^a^
		1996–2000 (130 patients) n, %	2001–2005 (287 patients) n, %	2006–2010 (439 patients) n, %	
Country					
Malaysia	445 (52.0)	92 (70.8)	130 (45.3)	223 (50.8)	NA
Singapore	411 (48.0)	38 (29.2)	157 (55.7)	216 (49.2)	
Age, years (median)	53	49	52	54	<0.001
Ethnicity					0.001
Chinese	508 (59.3)	72 (55.4)	186 (64.8)	250 (56.9)	
Malay	228 (26.6)	44 (33.8)	77 (26.8)	107 (24.4)	
Indian	92 (10.7)	12 (9.2)	20 (7.0)	60 (13.7)	
Other races	28 (3.3)	2 (1.5)	4 (1.4)	22 (5.0)	
Primary tumor size					0.087
5 cm and less	178 (29.3)	22 (20.2)	67 (32.8)	89 (30.3)	
More than 5cm	429 (70.7)	87 (79.8)	137 (67.2)	205 (69.7)	
Unknown	249	21	83	145	
Presence of T4 tumor					0.136
Yes	283 (39.0)	43 (35.2)	95 (35.8)	145 (42.9)	
No	442 (61.0)	79 (64.8)	170 (64.2)	193 (57.1)	
Unknown	131	8	22	101	
Estrogen receptor status					0.469
Positive	431 (59.8)	31 (53.4)	155 (62.0)	245 (59.3)	
Negative	290 (40.2)	27 (46.6)	95 (38.0)	168 (40.7)	
Unknown	135	72	37	26	
No of organs with distant metastasis					0.007
1	378 (48.7)	65 (60.2)	135 (48.6)	178 (45.6)	
2	249 (32.1)	30 (27.8)	99 (35.6)	120 (30.8)	
3 or more	149 (19.2)	13 (12.0)	44 (15.8)	92 (23.6)	
Unknown	80	22	9	49	
Visceral metastasis					0.001
Yes	675 (87.0)	82 (75.9)	243 (87.4)	350 (89.7)	
No	101 (13.0)	26 (24.1)	35 (12.6)	40 (10.3)	
Unknown	80	22	9	49	

^a^Continuous variables were tested using Kruskal Wallis test, while categorical variables were tested using Chi square test. *P* value less than 0·05 is considered statistically significant.

**Table 2:** Trends in Management of Patients with *De Novo* Metastatic Breast Cancer in an Asian Setting.

Characteristics	Overall (856 patients) N, %	Period of diagnosis			P value^a^
		1996–2000 (130 patients) n, %	2001–2005 (287 patients) n, %	2006–2010 (439 patients) n, %	
Surgery of primary tumor					0.001
No	534 (62.4)	86 (66.2)	189 (65.9)	259 (59.0)	
Mastectomy	301 (35.2)	36 (27.7)	90 (31.4)	175 (39.9)	
Breast conserving surgery	21 (2.5)	8 (6.2)	8 (2.8)	5(1.1)	
Surgical margins^b^					0.006
Free	187 (73.0)	13 (52.0)	43 (63.2)	131 (80.4)	
Positive	69 (27.0)	12 (48.0)	25 (36.8)	32 (19.6)	
Unknown	66	19	30	17	
Radiotherapy to breast/chest wall/other sites					0.126
Yes	220 (33.7)	24 (23.3)	65 (37.8)	131 (34.7)	
No	432 (66.2)	79 (76.7)	107 (62.2)	246 (65.1)	
Unknown	204	27	115	62	
Chemotherapy, n (%)					0.008
Yes	460(59.2)	71 (54.6)	109 (52.4)	280 (63.8)	
No	314(40.8)	59 (45.4)	99 (47.6)	156 (35.8)	
Unknown	82	0	79	3	
Endocrine therapy^c^					0.263
Yes	212 (59.1)	13 (44.8)	65 (59.6)	134 (60.6)	
No	147 (40.9)	16 (55.2)	44 (40.4)	87 (39.4)	
Unknown	74	2	46	26	

^a^Continuous variables were tested using Kruskal Wallis test, while categorical variables were tested using Chi square test. *P* value less than 0·05 is considered statistically significant.

^b^Only includes patients receiving surgery.

^c^Only includes patients with hormone receptor positive tumours.

**Table 3:** Cumulative relative survival of patients presenting with de novo metastatic breast cancer by period of diagnosis.

Period	Time	At diagnosis	Year 1	Year 2	Year 3
1996–2000	Numbers entering interval	130	66	33	24
	Relative survival, % (95% CI)	–	54.9 (45·7–63.1)	27.9 (20.2–36.0)	20.6 (13.9–28.2)
2001–2005	Numbers entering interval	287	155	108	76
	Relative survival, % (95% CI)	–	57.0 (50.8–62.7)	40.1 (34.2–45.9)	28.8 (23.4–34.3)
2006–2010	Numbers entering interval	439	278	174	83
	Relative survival, % (95% CI)	–	64.3 (59.6–68.7)	45.3 (40.5–50.1)	33.6 (28.8–38.5)

**Table 4:** Relative Risk of Mortality in Patients with *De Novo* Metastatic Breast Cancer by Period of Diagnosis.

Death	Period of diagnosis		
	1996–2000^a^	2001–2005	2006–2010
Relative excess risk (95% CI)^b^	1.0	0.86 (0.68–1.09)	0.68 (0.54–0.86)^h^
Relative excess risk (95% CI)^c^	1.0	0.82 (0.65–1.05)	0.68 (0.54–0.87)^h^
Relative excess risk (95% CI)^d^	1.0	0.82 (0.64–1.05)	0.66 (0.52–0.84)^h^
Relative excess risk (95% CI)^e^	1.0	0.82 (0.64–1.05)	0.71 (0.56–0.89)^h^
Relative excess risk (95% CI)^f^	1.0	0.87 (0.68–1.12)	0.77 (0.61–0.98)^h^
Relative excess risk (95% CI)^g^	1.0	0.89 (0.69–1.16)	0.79 (0.62–1.01)

^a^Reference period.

^b^Derived using Poisson regression model, adjusted for center, and follow-up time.

^c^Similar as model 2, and additionally adjusted for age at diagnosis, and ethnicity.

^d^Similar as model 3, and additionally adjusted for T stage at diagnosis, ER status, number of organs with metastatic involvement, and presence of visceral metastasis.

^e^Similar as model 4, and additionally adjusted for systemic therapy (chemotherapy, and hormone therapy).

^f^Similar as model 5, and additionally adjusted for surgery of primary tumor, surgical margins.

^g^Similar as model 6, and additionally adjusted for radiotherapy of breast, chest wall, and other metastatic sites.

^h^Statistically significant.
